# Emergency Decompressive Craniostomy “Burr Hole” Using an Intraosseous Vascular Access System in a Resource-Limited Setting: A Technical Report on a Cadaver

**DOI:** 10.7759/cureus.24420

**Published:** 2022-04-23

**Authors:** Jesse C Wu, Kevin Cao, Jeremy Mayfield, Latha Ganti

**Affiliations:** 1 Emergency Medicine, University of Central Florida College of Medicine, Orlando, USA; 2 Emergency Medicine, HCA Florida Osceola Hospital, Kissimmee, USA; 3 Internal Medicine, Einstein Medical Center Montgomery, East Norriton, USA; 4 Emergency Medicine, Envision Physician Services, Fort Lauderdale, USA

**Keywords:** epidural hematoma, extra-axial hemorrhage, intraosseous drill, craniostomy, burr hole

## Abstract

This paper describes an alternative approach to emergency burr hole evacuation of epidural hematoma using the intraosseous (IO) vascular access system. The IO vascular access system is commonly available in the Emergency Department. We demonstrate that it can be used in rare situations where immediate neurosurgical intervention and the standard cranial drill and brace are not available for burr hole craniostomy.

## Introduction

Extra-axial hemorrhage is bleeding that occurs within the skull but is outside of the brain parenchyma. Forms of extra-axial hemorrhage include both epidural hematomas (EDH) and subdural hematomas (SDH). These are often a result of traumatic brain injuries that necessitate immediate clinical decision-making in the emergency department (ED) to determine the severity and possible intervention by both emergency medicine and neurosurgery teams. Delay in assessment and possible intervention may result in an expanding hematoma, resulting in worsening sequelae secondary to increase in intracranial pressure (ICP).

EDH is a life-threatening condition when blood, commonly from a branch of the middle meningeal artery, rapidly accumulates in the potential space between the outer surface of dura and the inner surface of the skull, often secondary to traumatic brain injury (TBI). EDH accounts for approximately 10% of TBI with mortality ranging from 7% to almost 30% depending on the severity and whether timely neurosurgical intervention was achieved [1-2]. However, in the remote clinical setting, immediate neurosurgical intervention may not be available. Indications for emergent cranial burr hole in the ED include evidence of expanding EDH with midline shift on computed tomography (CT), deteriorating mental status with anisocoria, Glasgow Coma Scale < 8, and neurosurgery intervention not being available within a reasonable time [3].

SDH is another life-threatening form of bleeding that results in blood collection between the dura mater and arachnoid mater. Patients afflicted with TBI may have both SDH and EDH. Patients with severe TBI have acute SDH at a range of approximately 12% and 29% [4]. Emergent burr hole criteria for SDH are identical to that of EDH. SDHs typically have a more favorable outcome when compared to EDHs based on both severe and mild TBI [2]. Meticulous, but swift, decision-making is required to establish successful burr hole decompression. Decompression typically involves drilling a hole for egress.

Emergency cranial burr hole decompression is well described in the literature [5-10]. However, most of these techniques involve either a hand-held or electric cranial drill or a brace that is not commonly available in the ED. By contrast, the intraosseous (IO) vascular access system is routinely available in the ED for emergent vascular access. It can be an alternative option for emergent cranial burr hole procedures.

Currently, the study on the use of IO vascular access system in burr hole decompression is limited to case reports and cadaver studies [11-13]. In this technical report, we aim to illustrate a step-by-step approach to cranial burr hole decompression using an IO vascular access system on a cadaver.

## Technical report

**Figure 1:**
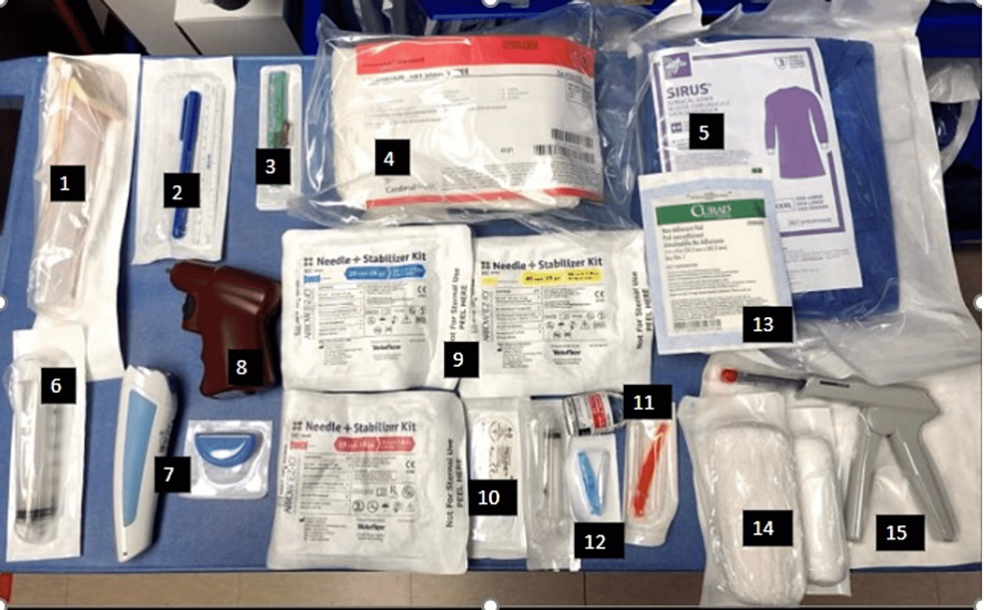
Burr hole supplies. 1) Chlorhexidine; 2) sterile marking pen with ruler; 3) number 10 scalpel; 4) laceration kit that includes sterile drapes, gauze, forceps, needle driver, scissor; 5) personal protective equipment, which includes but is not limited to sterile gown and gloves, surgical mask, and cap; 6) large syringe for aspiration of blood, preferably 30 cc-50 cc; 7) electric shaver with blade; 8) power driver for the intraosseous needle; 9) intraosseous vascular access system with three different size needles, 15 mm (pink), 25 mm (blue), 45 mm (yellow); 10) suture for skin closure; 11) lidocaine for local anesthesia before incision; 12) syringe and needles to draw up and inject lidocaine; 13 and 14) non-adherent gauze and roll gauze to dress the wound and secure the intraosseous needle in place after placement; 15) skin stapler as another option for skin closure.

Once the indication for an emergency burr hole has been determined, gather the necessary supplies (Figure 1).

**Figure 2:**
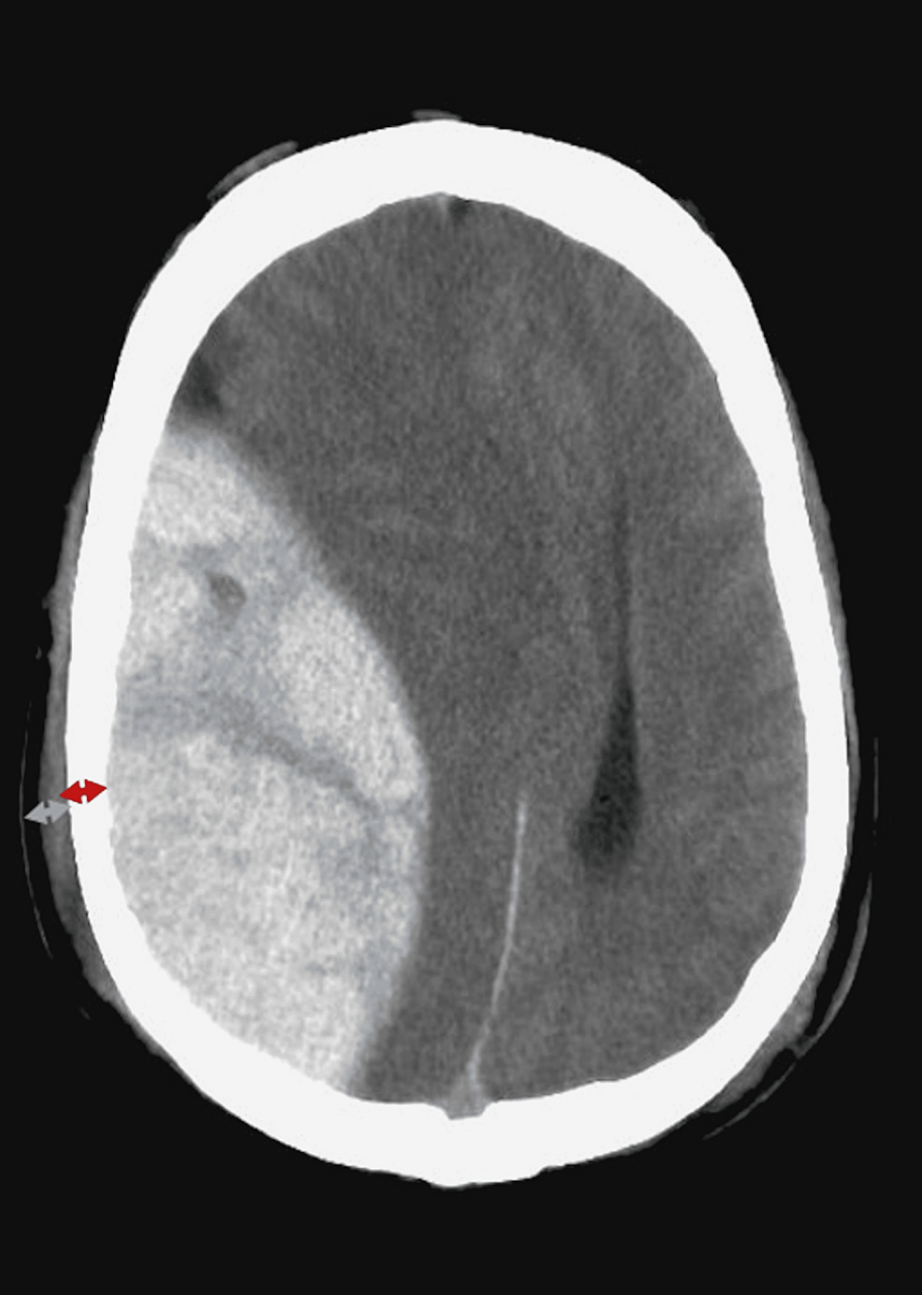
Non-contrast brain CT depicting epidural hematoma. The skull thickness (red arrow) should be measured in order to select the appropriately sized intraosseous needle to ensure skull penetration and to avoid injuring the parenchyma. If the scalp incision is not made, the scalp thickness (gray arrow) should also be taken into account when selecting the intraosseous needle. Case courtesy of Associate Professor Frank Gaillard, www.radiopaedia.org.

Ideally, the patient should be intubated and sedated. All team members should be briefed on the necessity and details of the procedure. Informed consent should be obtained as appropriate. This procedure should be discussed with and performed under neurosurgical guidance via telephone or video conferencing. Reviewing of the CT should be done to identify the anatomical location of the hemorrhage and measure the skull thickness to determine the depth of penetration and select the appropriate IO needle (Figure 2).

**Figure 3:**
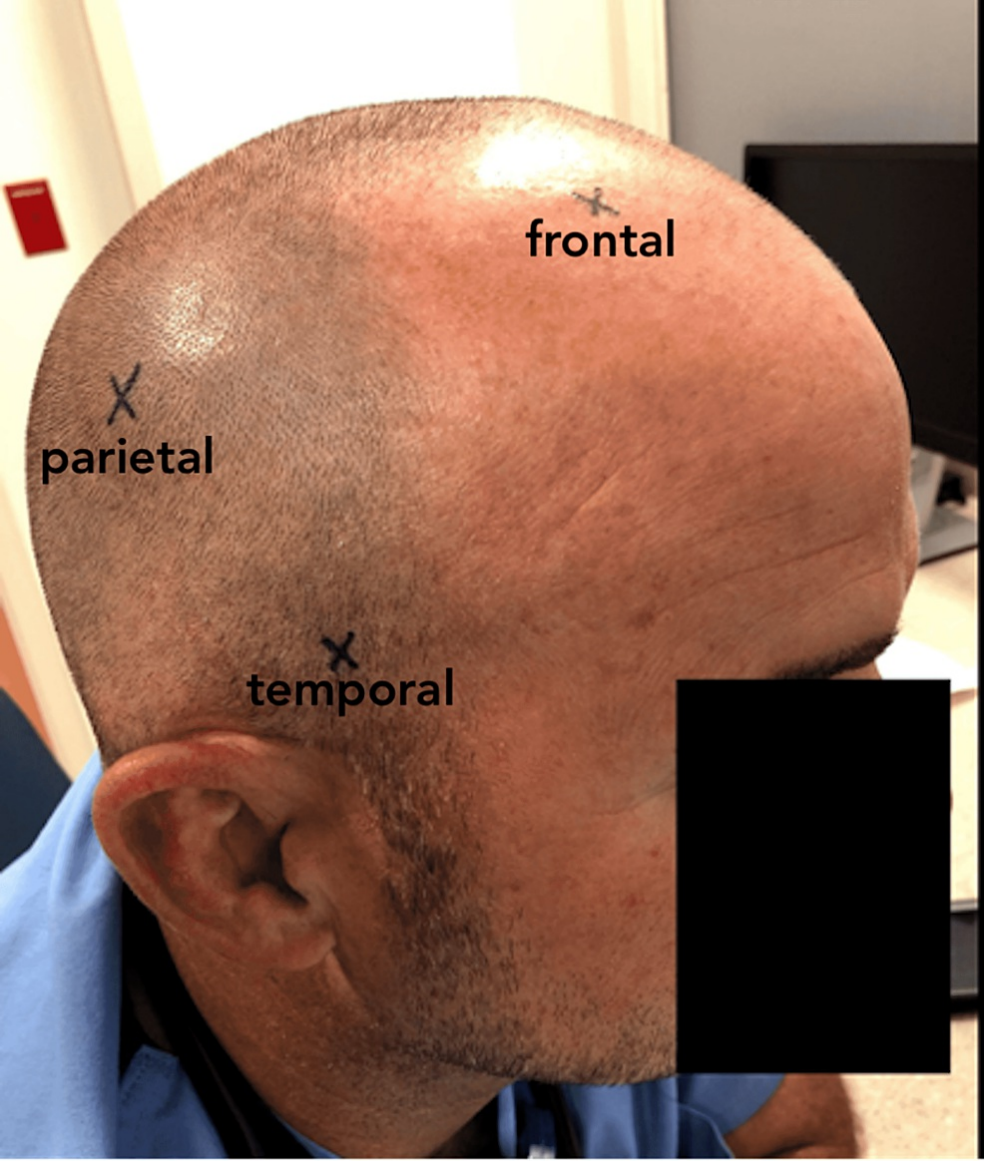
Clinical photograph demonstrating anatomical landmarks for the burr hole. Frontal: 10 cm above the eye in the mid-pupillary line. Temporal: 2 cm anterior and superior to the ear. Parietal: Over the parietal eminence. The subject in the photo gave written informed consent for this photograph.

The authors do not recommend proceeding with this procedure without CT confirmation of the hemorrhage. However, if CT is not available, the anatomical landmark is 2 cm anterior and 2 cm superior to the tragus on the ipsilateral side of the blown pupil (Figure 3).

**Figure 4:**
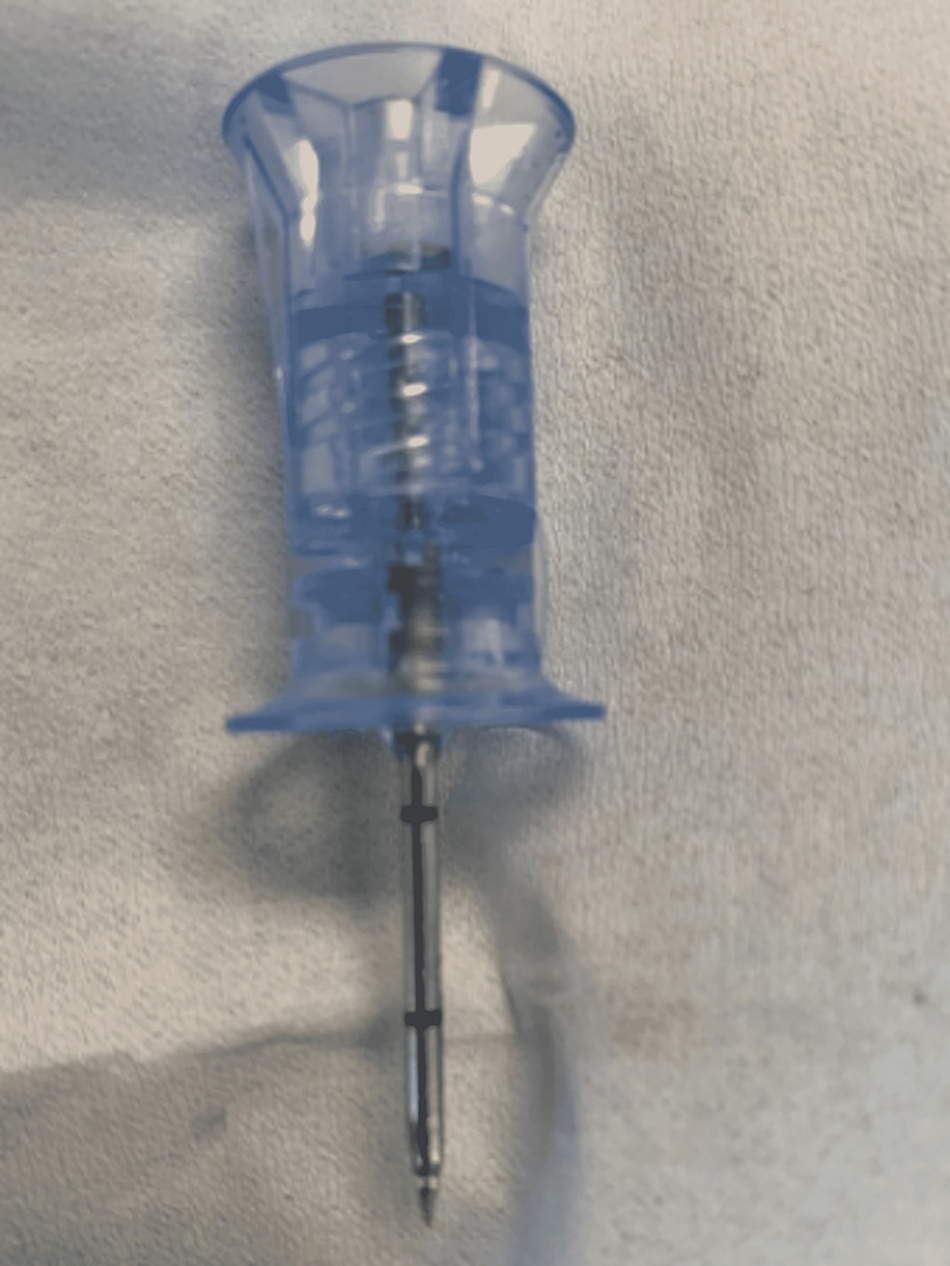
25 mm intraosseous needle.

The 25 mm IO needle is appropriate in most cases in the absence of CT measurement of skull thickness [12] (Figure 4).

**Figure 5:**
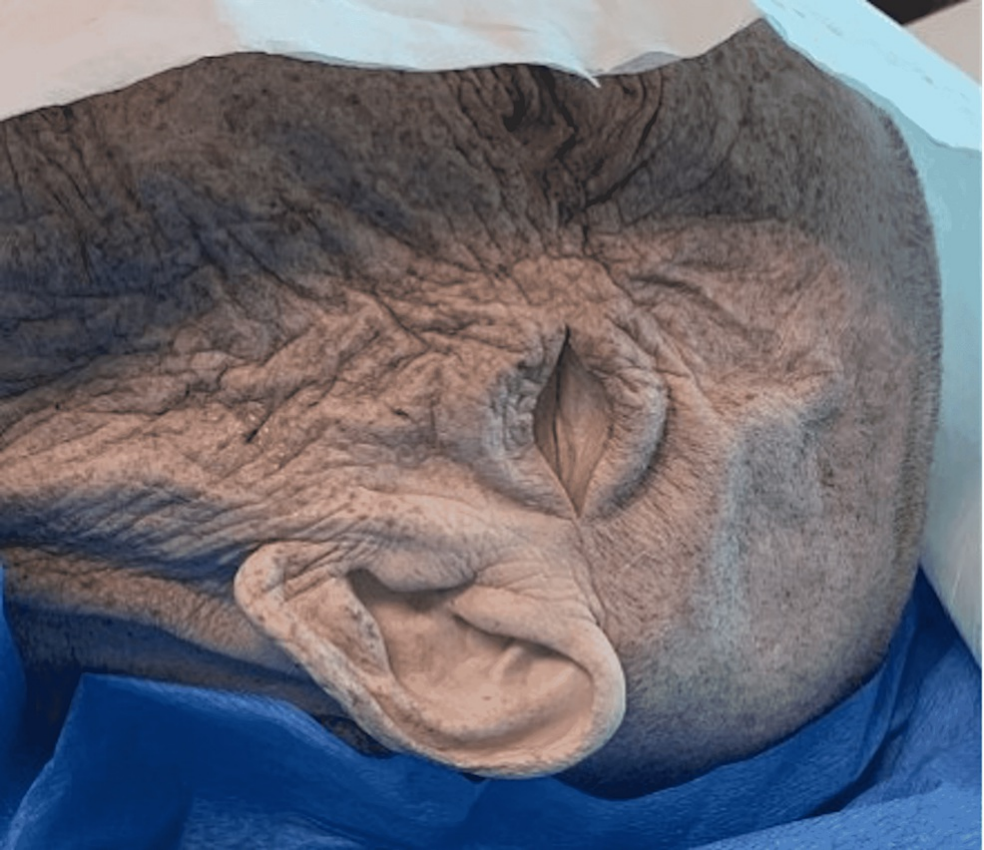
On this cadaver, a longitudinal incision 2 cm anterior and 2 cm superior to a tragus was made. Ideally, a craniocaudal incision should be made to avoid transecting the superficial temporal artery.

Other burr hole locations have also been described in the literature, such as the parietal burr hole over the parietal eminence and the frontal burr hole about 10 cm above the eye in the mid-pupillary line [13]. The temporal burr hole is described in this paper. Position the patient supine. The scalp should be shaved with an electric shaver or razor, sterilely prepped with betadine or chlorhexidine, and draped. The burr hole area should be marked so its center is over the center of the hemorrhage. Anesthetics such as lidocaine with epinephrine should be injected subcutaneously (approximately 3 ml) with a small-bore needle such as a 25 g. Make a 3-5 cm cranial to caudal incision down to the periosteum (Figure 5).

**Figure 6:**
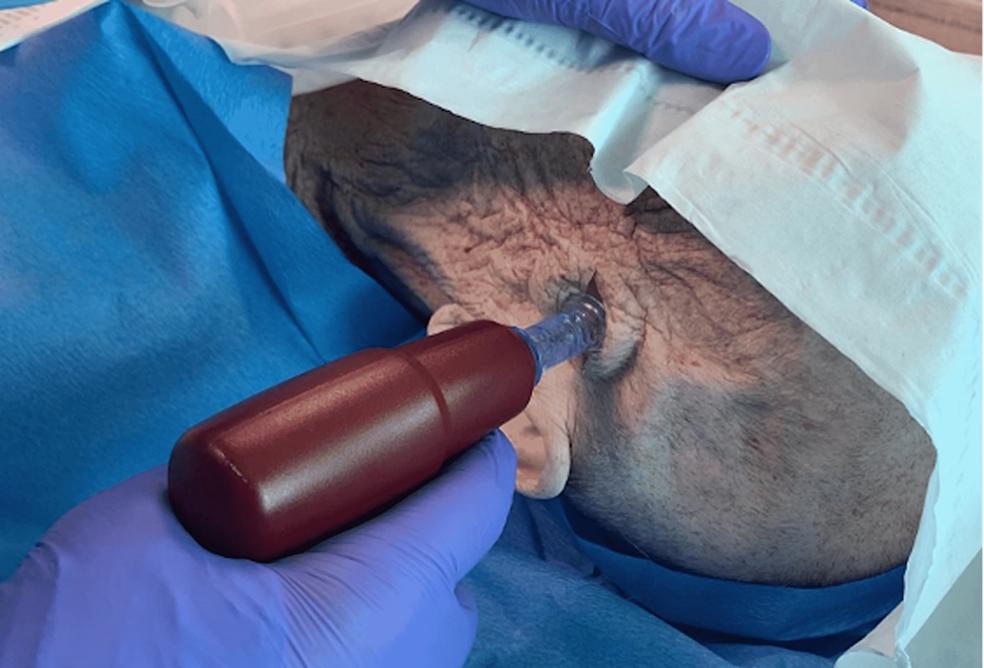
Advance the intraosseous (IO) needle with the power driver until the skull is penetrated, a sudden decrease in resistance should be felt.

Make an effort to identify the superficial temporal artery (STA) and stay anterior to it. If STA is transected, be ready for hemostasis. Expose the periosteum and use the self-retaining retractor if needed. Depending on skull thickness, load the appropriate IO vascular access system needle onto the power driver. Stabilize the head to prevent tilting during the procedure. Orient the needle perpendicular to the bone. Advance the needle with the power driver until there is a sudden decrease in resistance, twisting to remove the stylet (Figure 6).

**Figure 7:**
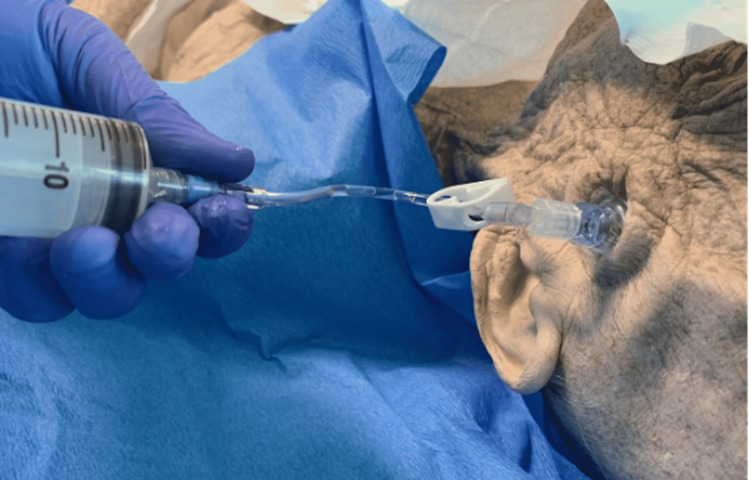
Remove the stylet after inserting the intraosseous (IO) needle, connect the extension tubing, and attach a large syringe to aspirate blood. Saline is used in this procedure to simulate fluid aspiration.

Apply the stabilizer dressing and connect the extension tubing. Connect a large syringe (30-50 ml) to the tubing and aspirate for blood (Figure 7).

Arrange for expedient transfer of the patient to definitive care at a facility with neurosurgery capability.

## Discussion

Evacuation of an expanding extra-axial hemorrhage is a time-sensitive intervention to prevent cerebral herniation. Increased time to evacuation is associated with worse outcomes in patients who have evidence of cerebral herniation and increased ICP, such as altered mental status, GCS <8, or anisocoria [14,15]. On the contrary, emergency decompressive craniostomy in the ED is associated with better outcomes in patients with evidence of cerebral herniation from the hemorrhage [16]. Ideally, medical interventions to lower the ICP should occur simultaneously, such as with the administration of mannitol or hypertonic saline.

The use of IO vascular access system in cranial burr holes has only been described in a few case reports and a cadaver study to date [11-13,17]. We believe our paper to be the first to describe this procedure in a step-by-step fashion in peer-reviewed literature. The IO vascular access system is readily available in most EDs and requires little training to use, and many emergency providers are already proficient with it. We have demonstrated that it is feasible to evacuate dangerous and compressive extra-axial hemorrhage with the IO needle in a resource-limited setting when no other safer or proven alternative is available to save the patient’s life.

Iatrogenic intraparenchymal injury, infection, misplacement of the IO needle, and hemorrhage are several of the potential complications of this procedure. Therefore, the risks and benefits of the procedures should be considered and discussed with neurosurgery, the patient, and the patient’s family as appropriate beforehand. This procedure should be done in a sterile fashion. However, sterility is difficult to maintain in the ED, especially in emergent procedures such as the cranial burr hole. Thus, antibiotics prophylaxis with Staphylococcus coverage should be administered with this procedure. To prevent misplacement of the IO needle, it is crucial to confirm skull thickness and the location of the hemorrhage on CT and select the appropriately sized IO needle. It is possible to perform this procedure without exposing the periosteum first. If this is the case, the thickness of the scalp should also be taken into account when selecting the IO needle to ensure the length of the needle can go through both the scalp and the skull. Note that it is likely that there could be some soft-tissue swelling overlying the intracranial hematoma, requiring a longer IO needle. A potential limitation of this procedure is the IO needle may not be able to aspirate blood clots, so this procedure is better suited for new hemorrhages or in patients who have been anticoagulated at baseline. However, even aspiration of a small volume of blood can lead to significant clinical improvement. More studies are needed to elucidate the technical aspects of this procedure such as whether it is possible to aspirate blood clots with the IO needle.

## Conclusions

This paper describes an alternative approach to the emergent decompressive craniostomy (burr hole) procedure using the IO vascular access system on a cadaver to potentially evacuate life-threatening extra-axial hemorrhage causing cerebral herniation. The authors want to highlight that this procedure is not standard practice. Using the IO vascular access system for cranial burr holes is an off-label use and should only be considered when it is necessary to save a life and neurosurgical intervention and standard equipment are not immediately available in a resource-limited setting. If this procedure is to be performed as a last resort to save a life, it should be done under neurosurgery guidance. The risks and benefits of the procedure should be considered and discussed with the team, family, and neurosurgery. More research is needed in this area to explore the safety, legal, and ethical considerations.

## References

[1] Khairat A, Waseem M (2021). Epidural Hematoma. https://pubmed.ncbi.nlm.nih.gov/30085524/.

[2] Aromatario M, Torsello A, D'Errico S, Bertozzi G, Sessa F, Cipolloni L, Baldari B (2021). Traumatic epidural and subdural hematoma: epidemiology, outcome, and dating. Medicina (Kaunas).

[3] Wilson MH, Wise D, Davies G, Lockey D (2012). Emergency burr holes: "How to do it". Scand J Trauma Resusc Emerg Med.

[4] Bajsarowicz P, Prakash I, Lamoureux J, Saluja RS, Feyz M, Maleki M, Marcoux J (2015). Nonsurgical acute traumatic subdural hematoma: what is the risk?. J Neurosurg.

[5] Ganti L (2016). Burr Hole Craniotomy. Atlas of Emergency Medicine Procedures.

[6] Howard A, Krishnan V, Lane G, Caird J (2020). Cranial burr holes in the emergency department: to drill or not to drill?. Emerg Med J.

[7] Motohashi O, Kameyama M, Shimosegawa Y, Fujimori K, Sugai K, Onuma T (2002). Single burr hole evacuation for traumatic acute subdural hematoma of the posterior fossa in the emergency room. J Neurotrauma.

[8] Habibi Z, Meybodi AT, Haji Mirsadeghi SM, Miri SM (2012). Burr-hole drainage for the treatment of acute epidural hematoma in coagulopathic patients: a report of eight cases. J Neurotrauma.

[9] Smith SW, Clark M, Nelson J, Heegaard W, Lufkin KC, Ruiz E (2010). Emergency department skull trephination for epidural hematoma in patients who are awake but deteriorate rapidly. J Emerg Med.

[10] Liu JT, Tyan YS, Lee YK, Wang JT (2006). Emergency management of epidural haematoma through burr hole evacuation and drainage. A preliminary report. Acta Neurochir (Wien).

[11] Bulstrode H, Kabwama S, Durnford A, Hempenstall J, Chakraborty A (2017). Temporising extradural haematoma by craniostomy using an intraosseous needle. Injury.

[12] Durnford S, Bulstrode H, Durnford A, Chakraborty A, Tarmey NT (2018). Temporising an extradural haematoma by intraosseous needle craniostomy in the District General Hospital by non-neurosurgical doctors - A case report. J Intensive Care Soc.

[13] Donovan DJ, Moquin RR, Ecklund JM (2006). Cranial burr holes and emergency craniotomy: review of indications and technique. Mil Med.

[14] Cohen JE, Montero A, Israel ZH (1996). Prognosis and clinical relevance of anisocoria-craniotomy latency for epidural hematoma in comatose patients. J Trauma.

[15] Haselsberger K, Pucher R, Auer LM (1988). Prognosis after acute subdural or epidural haemorrhage. Acta Neurochir (Wien).

[16] Nelson JA (2011). Local skull trephination before transfer is associated with favorable outcomes in cerebral herniation from epidural hematoma. Acad Emerg Med.

[17] Gustafson ML, Edwards J, Tager A (2021). Emergency Burr Hole utilizing the EZ-IO™ drill: A pilot cadaver study. Am J Emerg Med.

